# Hyperbaric oxygen therapy ameliorates acute brain injury after porcine intracerebral hemorrhage at high altitude

**DOI:** 10.1186/s13054-015-0976-8

**Published:** 2015-06-15

**Authors:** Hai-tao Zhu, Chen Bian, Ji-chao Yuan, Xiao-jun Liao, Wei Liu, Gang Zhu, Hua Feng, Jiang-kai Lin

**Affiliations:** Department of Neurosurgery, Southwest Hospital, Third Military Medical University, 30 Gaotanyan Street, Chongqing, 400038 China; Department of Military Psychology, College of Psychology, Third Military Medical University, 30 Gaotanyan Street, Chongqing, 400038 China

## Abstract

**Introduction:**

Intracerebral hemorrhage (ICH) at high altitude is not well understood to date. This study investigates the effects of high altitude on ICH, and examines the acute neuroprotection of hyperbaric oxygen (HBO) therapy against high-altitude ICH.

**Methods:**

Minipigs were placed in a hypobaric chamber for 72 h before the operation. ICH was induced by an infusion of autologous arterial blood (3 ml) into the right basal ganglia. Animals in the high-altitude ICH group received HBO therapy (2.5 ATA for 60 min) 30 min after ICH. Blood gas, blood glucose and brain tissue oxygen partial pressure (PbtO_2_) were monitored continuously for animals from all groups, as were microdialysis products including glucose, lactate, pyruvate and glutamate in perihematomal tissue from 3 to 12 h post-ICH.

**Results:**

High-altitude ICH animals showed significantly lower PbtO_2_, higher lactate/pyruvate ratio (LPR) and glutamate levels than low-altitude ICH animals. More severe neurological deficits, brain edema and neuronal damage were also observed in high-altitude ICH. After HBO therapy, PbtO_2_ was significantly increased and LPR and glutamate levels were significantly decreased. Brain edema, neurological deficits and neuronal damage were also ameliorated.

**Conclusions:**

The data suggested a more serious disturbance of tissue oxygenation and cerebral metabolism in the acute stage after ICH at high altitude. Early HBO treatment reduced acute brain injury, perhaps through a mechanism involving the amelioration of the derangement of cerebral oxygenation and metabolism following high-altitude ICH.

## Introduction

Intracerebral hemorrhage (ICH) is the second most common and the deadliest subtype of stroke, with a high mortality rate [1]. It represents between 10 and 15 % of all strokes that occur in the US, Europe and Australia, and between 20 and 30 % of all strokes that occur in Asian countries [2]. The 30-day mortality rate is approximately 40 %. Furthermore, ICH has a significant morbidity among individuals who survive, and only 20 % of survivors are independent at six months post-stroke.

A growing number of people travel to, or reside at altitudes higher than 2,500 m, such as the North American Rocky Mountains and Chinese Qinghai-Tibetan Plateau, due to social and economic development. About 140 million people live permanently in high-altitude regions and their health needs more public awareness. Acute mountain sickness, high-altitude cerebral edema and high-altitude pulmonary edema comprise the main components of high-altitude illnesses. However, less well known are the other conditions, chiefly neurological, that may arise completely outside the usual definition of altitude sickness [3]. Stroke is outside the focus of the majority, and remains a major disease that seriously threatens the health of the people living at high altitudes. Some of the few studies on this subject have reported that long-term residence at high altitudes was associated with a higher risk of stroke, almost 10 times greater than residence at low altitude [4, 5]. Moreover, stroke at a younger age occurs more frequently at high altitude than at low altitude [6]. Another study showed ICH to be the dominant subtype of high-altitude stroke, and it induced higher disability and mortality rates than at low altitude [7]. The etiology and clinical manifestations of high-altitude ICH are quite different from those of low-altitude ICH. Thus far, we know little about secondary brain injury and the pathophysiology related to high-altitude ICH; thus, effective therapy for this disease is also very limited.

Several cerebral monitoring techniques permit continuous monitoring of cerebral physiology, such as brain tissue oxygen partial pressure (PbtO_2_) and neurochemistry [8, 9]. Use of these modalities is beneficial to optimize brain oxygen utilization and metabolism in patients with acute brain injury. It may provide an extended window for the prevention, early detection and treatment of ongoing secondary neuronal injury, and help to improve outcomes after ICH [10, 11]. However, the changes in cerebral oxygenation and metabolism in the early stage of high-altitude ICH, and the differences between high-altitude and low-altitude ICH regarding these parameters are unknown.

Early hyperbaric oxygen (HBO) therapy mitigates blood–brain barrier disruption and facilitates angiogenesis after ICH. HBO therapy also reduces secondary hemorrhage after focal cerebral ischemia [12]. The amount of oxygen that is dissolved in plasma and tissues can be elevated dramatically via raising the pressure and oxygen content of inspired air. This may improve the aerobic and neurochemical milieu in the injured brain region [13]. Hypobaric hypoxia, a prime characteristic of high-altitude areas, is a risk factor for high-altitude stroke [5, 14]. Thus, treatment of high-altitude-induced hypoxemia using HBO is logical and necessary. However, the effects of HBO on acute brain injury induced by high-altitude ICH have not been reported. Currently the underlying mechanisms of HBO treatment, especially cerebral oxygenation and metabolism, are not well understood.

Previously, we established a porcine model of high-altitude ICH, and observed more severe tissue lesion and neurological deficit seven days after ICH at high altitude compared to low altitude [15]. In this study, one goal was to evaluate the differences between low-altitude and high-altitude ICH with regards to cerebral oxygenation and metabolism in the acute stage (within 12 h) of porcine ICH. The other goal was to evaluate the effects of early treatment with HBO on brain damage in high-altitude ICH via multimodality monitoring.

## Methods

### Animals

**Fig. 1:**
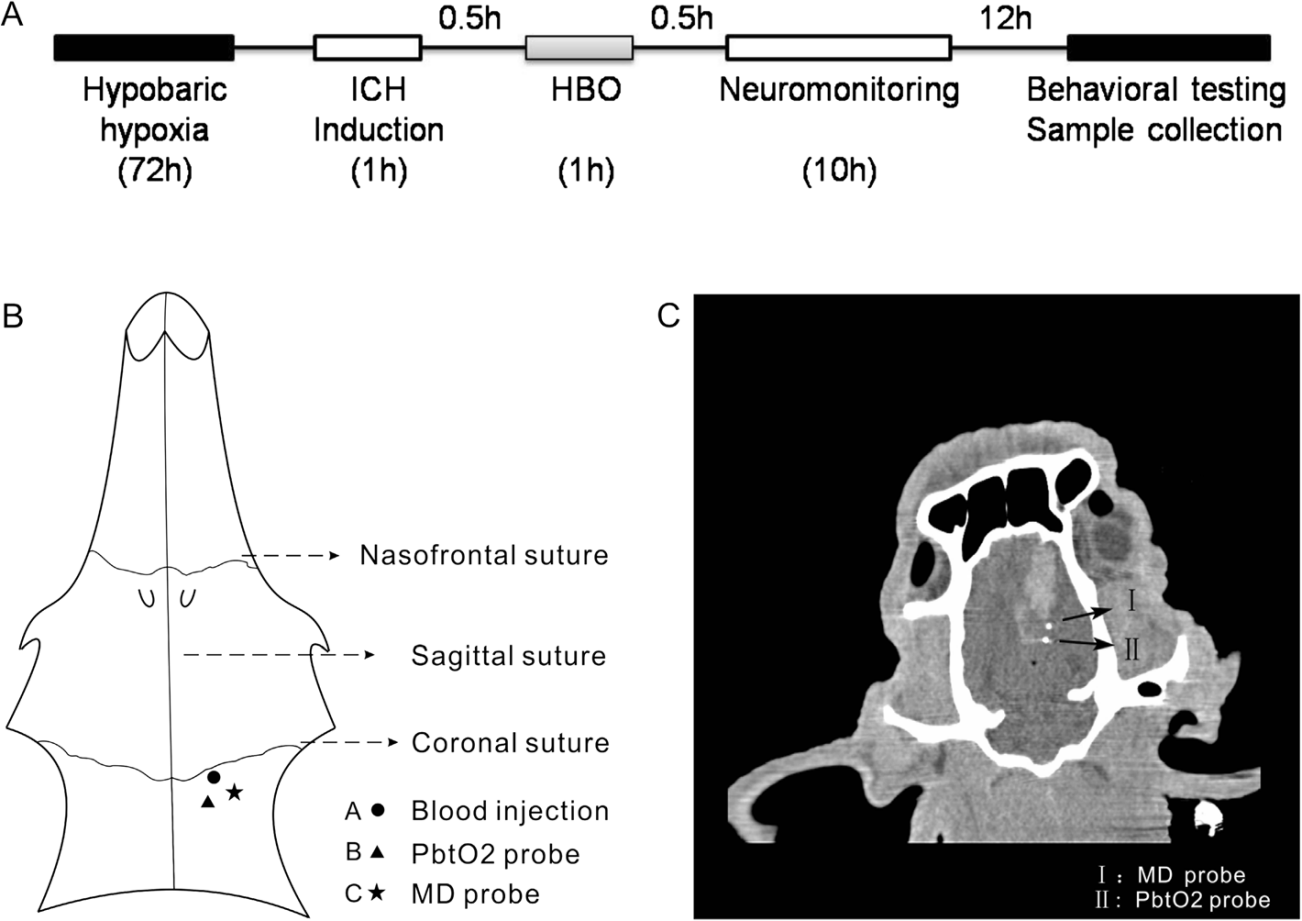
The ICH induction and the positioning of the probes. **a** The schematic representation of the experimental timeline. **b** Schematic drawing of catheter positioning relative to the injection site of the hematoma in the minipig cranium. **c** The relative position of the PbtO_2_ probe and MD probe in perihematomal tissue were confirmed using computed tomography

Forty-two male Guizhou Congjiang minipig (China) (15 to 20 kg) were obtained from the Experimental Animal Center of the Third Military Medical University, Chongqing, China. All experiments were conducted in accordance with animal care guidelines approved by the Animal Ethics Committee of the Third Military Medical University. The animals were housed with a 12-hour light/dark cycle and water and food provided *ad libitum*. They were randomly divided into five groups: plain (low-altitude) sham operation group (PS, six animals), plain blood-infusion group (PI, 10 animals), high-altitude sham operation group (HS, six animals), high-altitude blood-infusion group (HI, 10 animals) and high-altitude blood-infusion plus HBO therapy group (HI-HBO, 10 animals). PI, HI and HI-HBO animals received autologous arterial blood infusions; PS and HS animals received the same surgery but no blood infusions. The schematic representation of experimental timeline is shown in Fig. 1a.

### Animal surgical preparation

Pigs were initially anesthetized with ketamine (25 mg/kg Hengrui, Jiangsu, China) administered intramuscularly. After sedation, pentobarbital (30 mg/kg Xinya, Shanghai, China) was administered through an ear vein to achieve a deep surgical level of anesthesia. After placement of the femoral vein catheter, pentobarbital was infused at a rate of 10 mg/kg per hour until 12 h after ICH. Core temperature was measured with a rectal thermistor probe (Daxiong, Shenzhen, China). The right femoral artery was catheterized for mean arterial pressure (MAP) monitoring, and to permit the withdrawal of blood samples for determination of respiratory gases, acid–base status and glucose concentrations every three hours (Table 1).

**Table 1:** Physiological parameters in minipigs after ICH

	Hours post-ICH	PS (n = 6)	PI (n = 10)	HS (n = 6)	HI (n = 10)	HI-HBO (n = 10)
MAP (mmHg)	3	91.3 ± 2.5	93.8 ± 2.1	93.1 ± 2.6	94.2 ± 2.3	94.8 ± 2.2
	6	91.4 ± 2.4	94.1 ± 2.0	92.2 ± 2.6	92.8 ± 2.2	94.9 ± 2.2
	12	92.0 ± 2.5	93.0 ± 1.9	91.3 ± 2.4	93.7 ± 2.2	93.4 ± 2.0
Plasma glucose (mM)	3	4.45 ± 0.17	4.55 ± 0.13	4.48 ± 0.16	4.41 ± 0.12	4.49 ± 0.12
	6	4.55 ± 0.17	4.48 ± 0.13	4.46 ± 0.15	4.53 ± 0.12	4.48 ± 0.12
	12	4.35 ± 0.15	4.27 ± 0.12	4.36 ± 0.14	4.21 ± 0.11	4.38 ± 0.12
Core temperature (°C)	3	38.64 ± 0.06	38.73 ± 0.06	38.58 ± 0.07	38.70 ± 0.06	38.55 ± 0.08
	6	38.58 ± 0.06	38.66 ± 0.06	38.53 ± 0.06	38.61 ± 0.06	38.48 ± 0.07
	12	38.57 ± 0.05	38.60 ± 0.06	38.55 ± 0.06	38.53 ± 0.05	38.46 ± 0.06
pH	3	7.37 ± 0.02	7.37 ± 0.02	7.46 ± 0.03 ^a,b^	7.47 ± 0.02 ^a,b^	7.46 ± 0.02 ^a,b^
	6	7.38 ± 0.02	7.38 ± 0.01	7.46 ± 0.03 ^a,b^	7.45 ± 0.02 ^a,b^	7.48 ± 0.01 ^a,b^
	12	7.38 ± 0.02	7.39 ± 0.01	7.47 ± 0.03 ^a,b^	7.46 ± 0.02 ^a,b^	7.47 ± 0.02 ^a,b^
HCO_3_^−^ (mM)	3	24.50 ± 1.31	21.82 ± 1.09	18.18 ± 0.87 ^a,b^	18.53 ± 0.65 ^a,b^	18.46 ± 0.66 ^a,b^
	6	24.92 ± 1.27	22.03 ± 1.19	18.46 ± 0.84 ^a,b^	17.31 ± 0.76 ^a,b^	18.85 ± 0.62 ^a,b^
	12	23.87 ± 1.32	22.57 ± 1.17	17.97 ± 0.91 ^a,b^	17.73 ± 0.67 ^a,b^	18.64 ± 0.71 ^a,b^
SaO2	3	97.1 ± 0.5	96.4 ± 0.4	86.7 ± 0.9 ^a,b^	86.4 ± 0.6 ^a,b^	87.0 ± 0.7 ^a,b^
	6	97.8 ± 0.6	96.7 ± 0.5	87.3 ± 0.8 ^a,b^	85.9 ± 0.7 ^a,b^	86.8 ± 0.7 ^a,b^
	12	97.5 ± 0.6	97.1 ± 0.5	87.2 ± 0.8 ^a,b^	86.5 ± 0.6 ^a,b^	86.7 ± 0.6 ^a,b^
PaO_2_ (mmHg)	3	89.7 ± 1.5	84.5 ± 1.2	67.3 ± 1.4 ^a,b^	63.1 ± 0.9 ^a,b^	64.3 ± 1.0 ^a,b^
	6	90.7 ± 1.8	85.6 ± 1.1	68.3 ± 1.5 ^a,b^	63.6 ± 1.0 ^a,b^	63.9 ± 1.0 ^a,b^
	12	88.8 ± 1.5	88.4 ± 1.2	68.8 ± 1.5 ^a,b^	65.3 ± 1.0 ^a,b^	66.3 ± 1.2 ^a,b^
PaCO_2_ (mmHg)	3	35.1 ± 1.0	32.9 ± 0.6	25.1 ± 0.6 ^a,b^	23.5 ± 0.5 ^a,b^	24.8 ± 0.5 ^a,b^
	6	34.5 ± 0.9	33.8 ± 0.6	24.9 ± 0.6 ^a,b^	24.4 ± 0.6 ^a,b^	25.6 ± 0.6 ^a,b^
	12	33.9 ± 0.8	34.2 ± 0.7	25.4 ± 0.7 ^a,b^	24.1 ± 0.6 ^a,b^	25.1 ± 0.6 ^a,b^

At each time point examined, plasma glucose, MAP and core temperature showed no significant differences between the five groups. Values of blood gas analysis showed significant differences between the plain (low altitude) and high-altitude animals. There was no significant difference between the plain groups, and between the high-altitude groups. HI: high altitude blood infusion group, HI-HBO: high-altitude blood infusion plus HBO therapy group, HS: high altitude sham operation group, MAP: mean arterial blood pressure, PI: plain blood infusion group, PS: plain sham operation group

^a^ *P* <0.05, compared with PS; ^b^ *P* <0.05, compared with PI

### Intracerebral blood infusion

Surgery was performed as described previously, with moderate modifications [15, 16]. Each animal’s head was shaved and disinfected, and fully aseptic techniques were used. Three burr holes (2 mm in diameter) were drilled in the cranium: hole A (10 mm right, 2 mm posterior, for blood infusion), hole B (8 mm right, 8 mm posterior, for PbtO_2_ monitoring) and hole C (16 mm right, 8 mm posterior, for MD monitoring) (Fig. 1b). The right was relative to the sagittal suture, and the posterior was relative to the coronal suture. A 22-gauge sterile intravenous catheter (0.8 mm diameter, 25 mm long, Jierui, Weihai, China) was placed stereotaxically into the right basal ganglia (25 mm below the skull) and fixed firmly to the skull. Then, blood (1 ml) extracted from the right femoral artery was injected slowly into brain tissue through the inner cannula. A rate of 0.2 ml/min was controlled using a high-pressure syringe pump (Longerpump, Hebei, China). After blood infusion, the needle core was inserted into the inner cannula and maintained for 10 min. Next, the needle core was extracted and another 2 ml blood was injected slowly at the same rate. The needle core then was reinserted and maintained for 20 min, before the skull holes were sealed with bone wax.

### Experimental hypobaric hypoxia

To simulate hypobaric hypoxia, a hypobaric chamber (Moon, Yantai, China) was used. HS, HI and HI-HBO animals were placed in the temperature-controlled hypobaric chamber (20 ± 1 °C) for 72 h; the normal air in the chamber was adjusted to 0.6 ATA pressure, 14.5 % oxygen content (simulating an altitude of 4,000 m). The chamber was opened twice a day (for approximately 30 min each day) for feeding and cleaning. We slowly increased the air pressure in the chamber until it reached the normal atmospheric pressure of our laboratory (about 15 min) before opening the door of the chamber. After feeding and cleaning, we closed the door and then slowly decreased the air pressure in the chamber until the pressure was stabilized at 0.6 ATA (also about 15 min). Three days later, the minipigs received the same surgery as the PI group described above, and were kept in the hypobaric chamber with 0.6 ATA after ICH. During the entire experiment, an investigator was present to observe the animals in the chamber.

### Hyperbaric oxygen therapy plan

A hyperbaric pure oxygen chamber (Moon, Yantai, China) was used. HI-HBO animals were treated once with HBO therapy 30 min after ICH, with 2.5 ATA pressure and 100 % O_2_, boosting pressure for 15 min, stable pressure for one hour and reducing pressure for 15 min. Then they were sent to the hypobaric chamber (0.6 ATA) until 24 h after ICH. The HS and HI animals were always maintained in the hypobaric chamber for the end time point, and the animals in the PS and PI groups were only supplied air at normal pressure.

### Multimodal monitoring

The PbtO_2_ probe (Lincox, Ratingen, Germany) and microdialysis (MD) probe (CMA, Kista, Sweden) were inserted and fixed firmly two hours after ICH. The positions of the PbtO_2_ probe and MD probe in the perihematomal tissue were confirmed via computed tomography, and these probes were very close to the hematoma (Fig. 1c). The data were collected one hour after insertion due to the unreliability of readings in the initial period of time post-insertion. Then, these parameters were continuously recorded from three to 12 h after ICH. Microdialysates were perfused with a rate of 0.3 μl/min using an MD pump (CMA, Kista, Sweden), and samples were taken every hour. The MD vials were collected and immediately refrigerated at −80 °C. All vials were analyzed after the experiment using an MD automatic analyzer (CMA 600, Kista, Sweden).

### Neurobehavioral evaluation

Behavior was assessed in each group 24 h after surgery using the Purdy neurological deficit scale with moderate modification [17]. The scale included tests of motor function (0 to 5), consciousness (0 to 4), head turning (0 to 1), circling (0 to 1) and hemianopsia (0 to 1). Neurobehavioral evaluation was graded on a scale of 0 to 12 (completely normal score, 0; maximal deficit score, 12). Tests were conducted by an observer blinded to the groups.

### Brain tissue water content

At 24 h after surgery, half of the animals in each group were deeply anesthetized, and their skull was opened. Two brain tissue samples around the hematoma of each brain were cut and weighed. These samples were put into an oven at 100 °C for 72 h until a constant weight was reached. Water contents were expressed as a percentage of wet weight.

### Nissl staining

The other half of the animals of each group were anesthetized, and their brains were perfused *in situ* with 10 % formalin. Then, the brains were soaked in 4 % paraformaldehyde solution at 4 °C for 72 h. Brain tissue adjacent to the hematoma was cut, embedded in 30 % paraffin and cut into 20-μm thick sections. These sections were deparaffinized with xylene and graded alcohol. Toluidine blue was used according to a standard protocol. Each section was observed and photographed under an optical microscope (Olympus, Tokyo, Japan).

### Terminal deoxynucleotidyl transferase dUTP nick-end labeling staining

The terminal deoxynucleotidyl transferase dUTP nick-end labeling (TUNEL) assay was performed using the In situ Cell Death Detection Kit (Roche Molecular Biochemicals, Mannheim, Germany) that labels DNA strand breaks with fluorescein isothiocyanate according to a standard protocol. Each section was observed and photographed under a confocal microscope (Carl Zeiss, Oberkochen, Germany). Negative controls were obtained by omitting the TdT enzyme. The percentage of TUNEL-positive cells was expressed as the number of TUNEL-stained nuclei divided by the total number of 4′,6-diamidino-2-phenylindole (DAPI)-stained nuclei.

### Statistical analysis

All data were expressed as the means ± SEM. A model of repeated-measures analysis of variance (ANOVA) with *post-hoc* Bonferroni correction was used for analysis of various physiological parameters. One-way ANOVA with *post-hoc* Fisher’s least significant difference test was used for neurological scores and brain edema. All analyses were calculated using PASW software version 18 (SPSS, Chicago, USA). Statistical significance was preset at *P* <0.05.

## Results

### Tissue oxygenation in the perihematomal tissue

**Fig. 2:**
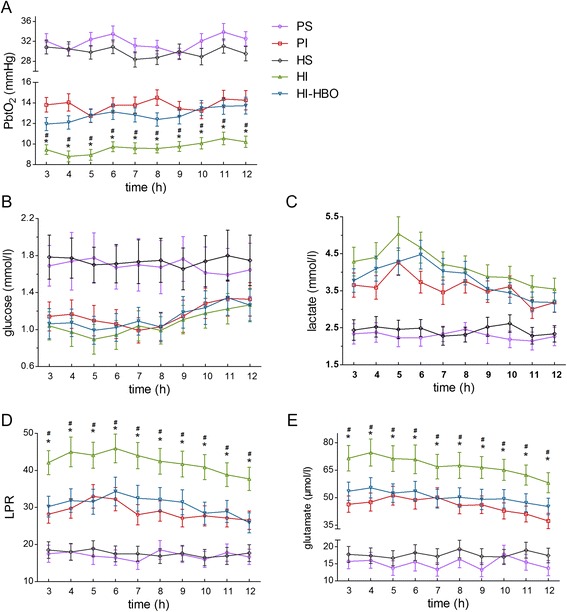
Changes in cerebral oxygenation and metabolism from three to 12 h post-ICH. **a** At each time point, PbtO_2_ in HI animals was significantly lower than in PI animals, and it was significantly elevated in HI-HBO animals. **b** Although the level of glucose in HI animals was lower than in the PI or HI-HBO animals at most of the time points, there was no significant difference between the three groups. **c** Likewise, lactate in HI animals was higher than in the PI or HI-HBO animals at all time points, but no significant difference existed between them. **d** LPR in HI animals was significantly higher than in the PI and HI-HBO animals at each time point. **e** Likewise, glutamate in HI animals was significantly higher than in the PI and HI-HBO animals at each time point. ^#^ *P* <0.05, compared with PI; ^*^ *P* <0.05, compared with HI-HBO

All minipigs survived and were observed to the end of the experiment. Although PbtO_2_ in the HS animals was lower than in the PS animals at most of the time points, no significant difference existed between these two groups (*P* >0.05). At each time point, PbtO_2_ in the PI, HI and HI-HBO animals was significantly lower than in the PS and HS animals. Furthermore, PbtO_2_ in the HI animals was significantly lower than in the PI animals and the HI-HBO animals at each time point (*P* <0.05). However, there were no significant differences between the PI and HI-HBO animals at any time point. PbtO_2_ at 11 or 12 h was significantly higher than at three hours in the HI-HBO animals (Fig. 2a).

### Cerebral metabolism in the perihematomal tissue

There was no significant difference between the PS and HS animals in glucose, lactate, lactate/pyruvate ratio (LPR) and glutamate (*P* >0.05). At each time point, glucose in the PI, HI and HI-HBO animals was significantly lower than in the PS and HS animals (*P* <0.05). Although glucose in the HI animals was lower than in the PI and HI-HBO animals at most of the time points, no significant difference was observed among the three groups (Fig. 2b). Lactate was significantly higher at five or six hours than at 12 h in the HI animals, and it was significantly higher at six hours than at 11 or 12 h in the HI-HBO animals. At each time point, lactate in the PI, HI and HI-HBO animals was significantly higher than in the PS and HS animals (*P* <0.05). Although lactate in the HI animals was higher than in the PI and HI-HBO animals at most of the time points, there was no significant difference among the three groups (Fig. 2c). It was significantly higher at six hours than at 12 h in the HI and HI-HBO animals about LPR. At each time point, LPR in the PI, HI and HI-HBO animals was significantly higher than in the PS and HS animals (*P* <0.05). Furthermore, LPR in the HI animals was significantly higher than in the PI and HI-HBO animals (*P* <0.05). However, there was no significant difference between the PI and HI-HBO animals (Fig. 2d). Glutamate was significantly higher at four hours than at 12 h in the HI and HI-HBO animals. It was also significantly higher in the PI, HI and HI-HBO animals than in the PS and HS animals (*P* <0.05). Furthermore, it was significantly higher in the HI animals than in the PI and HI-HBO animals at each time point (*P* <0.05). No significant difference existed in the PI and HI-HBO animals (Fig. 2e).

### Neurological deficits and brain edema

**Fig. 3:**
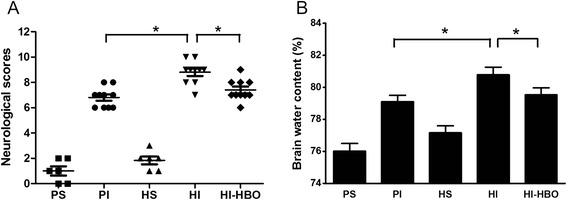
Changes in neurological functioning and brain edema 24 h after ICH. **a** High-altitude ICH showed more serious neurological function impairments than low-altitude ICH. However, there was no significant difference between HI and HI-HBO animals. **b** High-altitude ICH also showed more serious brain edema than low-altitude ICH. Moreover, brain water content in HI-HBO animals significantly decreased. ^*^ *P* <0.05

There was no significant difference between the PS and HS animals in neurological scores or brain water content. High-altitude ICH showed more serious impairments in neurological functioning than low-altitude ICH (*P* <0.05). Further, there was significant improvement in neurological deficits after HBO treatment at 24 h post-injury (Fig. 3a). High-altitude ICH also showed higher brain water content than low-altitude ICH (*P* <0.05). After HBO treatment, brain water content was significantly decreased 24 h after ICH (Fig. 3b).

### DNA damage and neuronal damage in perihematomal tissue

**Fig. 4:**
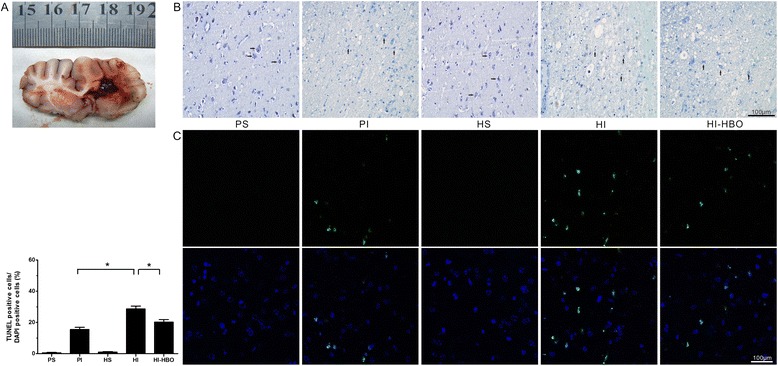
Nissl and TUNEL staining of the perihematomal tissue 24 h after ICH. **a** Coronal brain slice shows oval-shaped hematomas at the right basal ganglia. **b** Nissl staining showing a decrease in the number of stained cells and more severe structural damage of the neurons around the hematoma in HI animals. However, the neuronal damage in HI-HBO animals was reduced. Right arrow indicates normal neuron, up arrow indicates damaged neuron in the photos. **c** Quantification analysis indicating TUNEL-positive cells increased in HI animals and decreased in HI-HBO animals. The percentage of TUNEL-positive cells was expressed as the number of TUNEL-stained nuclei divided by the total number of DAPI-stained nuclei. ^*^ *P* <0.05. Bar = 100 μm

At 24 h post-ICH, coronal brain slices showed oval-shaped hematomas at the right basal ganglia (Fig. 4a). PS and HS animals showed lots of stained neurons with normal structure in Nissl staining. A decrease in the number of stained cells, as well as unclear or damaged structure in most of the neurons around the hematoma, were shown in PI animals. Furthermore, the decrease in the number of stained cells and structural damage of the neurons was more severe in the HI animals when compared with the PI animals. However, the neuronal damage in the HI-HBO group was less than that in the HI group (Fig. 4b). In addition, similar results for DNA damage were found with TUNEL staining. TUNEL-positive cells were evident in the perihematomal tissue at 24 h post-ICH. The number of TUNEL-positive cells around the hematoma increased dramatically in the HI group compared with the PI group (*P* <0.05). Significantly fewer TUNEL-positive cells were found in the HI-HBO group (Fig. 4c).

## Discussion

High-altitude ICH has been a somewhat obscure clinical problem to date, and there is a severe lack of experimental animal research into high-altitude ICH. In this animal study, high-altitude ICH animals manifested lower PbtO_2_, higher levels of LPR and glutamate, and more severe brain lesion and neurological outcomes than low-altitude ICH. HBO treatment (2.5 ATA for 60 min) 30 min after high-altitude ICH improved cerebral physiology, pathological damage and neurological deficit. One possible pathophysiological mechanism whereby HBO treatment attenuates brain injury is the modulation of cerebral oxygenation and metabolism during experimental high-altitude ICH.

PbtO_2_, the partial pressure of oxygen in the extracellular fluid of the brain tissue, reflects the availability of oxygen for oxidative energy production. Although PbtO_2_ does not directly assess cerebral blood flow, it is a complex and dynamic parameter representing the interaction between the delivery and demand of brain oxygen, as well as oxygen diffusion gradients [11]. PbtO_2_ monitoring can be used to identify cerebral perfusion pressure targets for optimal brain tissue oxygenation, and low perihematomal PbtO_2_ correlates with poorer outcomes after ICH [10]. In this study, high-altitude ICH showed lower PbtO_2_ than low-altitude ICH in the acute stage, and no significant difference in MAP existed between these groups. In addition to MAP, changes in arterial oxygenation can influence PbtO_2_ following ICH [18]. Therefore, the low PaO_2_ in response to hypobaric hypoxia may be an important cause of the worse cerebral oxygenation post-ICH. The observation that lower PbtO_2_ occurred in animals exposed to hypobaric hypoxia rather than normoxia after head trauma was consistent with our findings [19].

Despite some indicators of hypoperfusion, true ischemia in the perihematomal region was difficult to prove because of disproportionate reductions in the cerebral metabolic rate for oxygen and preserved oxygen extraction fractions in the perihematomal tissue [20]. A switch of concept from ischemic to metabolic penumbra was suggested recently. The transient focal increases in glucose metabolism have been interpreted as signs of ongoing neuronal injury in the metabolic penumbral tissue [21]. One or more non-ischemic mechanisms may contribute to this change in energy metabolism, including neuroinflammation, cytotoxicity or seizure activity. The interstitial glucose concentration is attributed to the balance between delivery from the blood capillaries and the cellular uptake. A low level of glucose was associated with worse outcome after brain injury [10]. In this study, a decrease in interstitial glucose concentration in the early phase, which was interpreted as an increase in glucose consumption, was observed after ICH. However, hypobaric hypoxia did not result in markedly lower glucose than normoxia post-ICH. One reason may be that hypoxia induces an increase in cerebral glucose concentrations in response to increased glucose transporters in brain microvessels and blood-to-brain glucose influx [22]. Another may be regional-specific changes in the cerebral glucose metabolism exposed to hypobaric hypoxia [23, 24]. Additionally, the cerebral glucose consumption may reach maximal capacity, so the glucose level is maintained at its lowest level possible. Serum glucose levels can affect cerebral glucose metabolism, so systemic glucose variability may be associated with poor outcome in critically ill patients [25]. However, interference in cerebral metabolism from serum glucose was low in this study due to the similar levels of serum glucose in each group.

In addition to reduced extracellular glucose, elevated lactate is also observed in some brain injury studies [26]. Additionally, lactate accumulations or pyruvate decreases in the extracellular space lead to an increase in LPR, which reflects the oxidative metabolism within the cytoplasm. In this study, cerebral lactate and LPR increased and were sustained until the end time point. Furthermore, patients after surgical evacuation of a hematoma with a poor outcome had significantly higher lactate and LPR than patients with a good outcome [27]. Although cerebral lactate may increase at high altitude [28, 29], lactate did not dramatically elevate after ICH at high altitude compared to low altitude in our study. We speculate that lactate may be used as an alternate energy source for neurons, potentially via shuttling of glycolytically derived carbons from astrocytes, especially in hypoxia [30]. Another ICH study reported that LPR and intracranial pressure decreased after mannitol administration despite a lack of increase in MD glucose, pyruvate and PbtO_2_ [31]. Recently, LPR has been interpreted as energy metabolic distress in the absence of ischemia, which possibly results from mitochondrial dysfunction, seizures or reduced substrate availability. In the present study, larger increases in LPR induced by hypobaric hypoxia indicate worse energy metabolism dysfunction at high altitude than at low altitude in the early stages post-ICH. Increased LPR in the bronchoalveolar fluid was also observed in high-altitude lung injury [32].

Elevated glutamate levels in the perihematomal region after ICH have been demonstrated in some low-altitude studies [33], and our data of glutamate are consistent with this finding. Glutamate activation of NMDA or AMPA receptors can result in increased glucose metabolism in perihematomal tissue after ICH [34]. Furthermore, our results suggest a higher accumulation of glutamate around the hematoma soon after high-altitude ICH. The expression change in glutamate receptors, such as NR1 and GluR2, mediates glutamate excitotoxicity in chronic hypobaric hypoxia [35]. These observations of multi-parameters show the complex features of brain metabolism as a function of substrate delivery, transport and consumption in the variable demand of the injured brain, especially at high altitude. Any interpretation of one single factor is limited by virtue of its intricate nature. Thus, more severe brain oxygenation reduction, energy metabolism dysfunction and glutamate accumulation occur jointly in the early stages of high-altitude ICH characterized by hypobaric hypoxia. Direct or indirect actions of cerebral oxygenation and energy metabolism distress, as well as glutamate excitotoxicity, synergistically elicit the death and degeneration of neural cells, which aggravates brain edema and cerebral damage after high-altitude ICH.

HBO has been widely used as the primary therapy in patients with decompression sickness, carbon monoxide poisoning and arterial gas embolism. Plenty of studies suggest that HBO treatment is effective in brain injury at low altitude. Early HBO therapy mitigates blood–brain barrier disruption and suppresses the progression of brain edema post-ICH by preventing occludin degradation and matrix metalloproteinase-9 activation in the perihematomal tissue [36]. In a murine ischemic stroke study, altered striatal energy metabolites and glutamate can be regulated by HBO, which might contribute to the neuroprotection of HBO therapy [37]. Additionally, normobaric hyperoxia also increases brain tissue oximetry, with a variable effect on lactate and LPR in severe traumatic brain injury patients [38]. Another study reported that HBO improved PbtO_2_ and metabolic distress, and had a more robust post-treatment effect than normobaric hyperoxia on oxidative cerebral metabolism, related to its ability to produce a PbtO_2_ of 200 mmHg or higher [13]. After early HBO treatment, an increase in PbtO_2_ may improve cerebral metabolism, cerebrovascular autoregulation, cerebral blood flow, neuroinflammation and brain edema. In turn, improvements in these can raise cerebral oxygenation. In this study, early HBO therapy also markedly elevated PbtO_2_ for 12 h in hypobaric hypoxia. A study about high-altitude brain injury also reported that HBO treatment can increase PbtO_2_ and regional cerebral blood flow 24 h after brain injury and benefit recovery [19]. HBO treatment following high-altitude ICH alleviated secondary brain injury and neurological deficits, which was attributed to ameliorated brain tissue oxygenation, energy metabolism dysfunction and glutamate excitotoxicity. HBO therapy has also been applied in many other diseases at high altitude. One hour of treatment in a portable hyperbaric chamber leads to a short-term improvement in symptoms of acute mountain sickness [39]. Improvements in the focal oxygen supply by HBO ameliorated calcific uremic arteriolopathy in high-altitude areas [40]. Additionally, high-altitude pulmonary edema and lung injury can be reduced by an induction of heat-shock protein 70 in the lung in response to HBO preconditioning [41].

There are some limitations to this study. First, maintenance in a hypobaric chamber for three days may be too short to totally emulate the long duration of ICH formation at high altitude. However, it must be noted that the current study was intended to be a hypothesis-generating pilot study. The absence of intracranial pressure and cerebral perfusion pressure monitoring is another limitation. Additionally, because our autologous blood injection model does not mimic small vessel rupture in ICH, microvascular breakdown effects are difficult to assess. Furthermore, the size of the sample is small and a larger number is needed for further investigation.

## Conclusions

Our findings indicate more serious disturbance of tissue oxygenation and cerebral metabolism, as well as worse brain pathology and neurological outcomes, in high-altitude ICH in comparison to low-altitude ICH in the acute stages in our porcine model. Early HBO treatment reduced brain edema and tissue damage via amelioration of cerebral oxygenation and metabolism, and this may be an important mechanism whereby HBO attenuates secondary brain injury following high-altitude ICH. All the data support modulation of tissue oxygenation and cerebral metabolism in perihematomal tissue as a potential therapeutic target, and suggest that early HBO treatment may be beneficial for ICH at high altitude.

## Key messages

- High-altitude ICH shows more serious disturbance of tissue oxygenation and cerebral metabolism in comparison to low-altitude ICH in the acute stages.
- Early HBO treatment reduced acute brain injury through a mechanism involving the amelioration of the derangement of cerebral oxygenation and metabolism following high-altitude ICH.

## Abbreviations

ATA: absolute atmosphere
HBO: hyperbaric oxygen
HI: high altitude blood infusion group
HI-HBO: high-altitude blood infusion plus
HBO: therapy group
HS: high altitude sham operation group
ICH: Intracerebral hemorrhage
LPR: lactate/pyruvate ratio
MAP: mean arterial pressure
MD: microdialysis
PbtO2: brain tissue oxygen partial pressure
PI: plain blood infusion group
PS: plain sham operation group
